# Glassy Carbon Electrode Modified with N-Doped Reduced Graphene Oxide Sheets as an Effective Electrochemical Sensor for Amaranth Detection

**DOI:** 10.3390/ma15093011

**Published:** 2022-04-21

**Authors:** Hediyeh Moradpour, Hadi Beitollahi, Fariba Garkani Nejad, Antonio Di Bartolomeo

**Affiliations:** 1 Department of Chemistry, Graduate University of Advanced Technology, Kerman 7631885356, Iran; hediyehmoradpour2020@gmail.com (H.M.); f.garkani95@gmail.com (F.G.N.); 2 Environment Department, Institute of Science and High Technology and Environmental Sciences, Graduate University of Advanced Technology, Kerman 7631885356, Iran; 3 Department of Physics “E.R. Caianaiello”, University of Salerno, 84084 Fisciano, Salerno, Italy

**Keywords:** reduced graphene oxide, electrochemical sensor, amaranth, N-doped reduced graphene oxide, glassy carbon electrode

## Abstract

Amaranth is one of the synthetic azo colorants used to improve the appearance and to increase the appeal of some foods and soft drinks. The excessive consumption of amaranth can be associated with health side effects, emphasizing the need to monitor this food dye. Accordingly, the present study aimed to introduce an electrochemical sensor of glassy carbon electrode (GCE) modified with N-doped reduced graphene oxide (N-rGO), N-rGO/GCE, to detect the amaranth sensitively and rapidly. Several electrochemical techniques such as differential pulse voltammetry (DPV), linear sweep voltammetry (LSV), chronoamperometry (CHA), and cyclic voltammetry (CV) are exploited for the evaluation of the efficiency of the developed electrode for the detection of amaranth. We found that N-rGO/GCE enhanced amaranth oxidation, thus significantly elevating the current signal. Amaranth showed that calibration curves ranged from 0.1 to 600.0 µM, and the limit of detection (LOD) (S/N = 3) was 0.03 μM. Finally, the developed sensor was effectively applied for real samples (tap water, apple juice, and orange juice) with acceptable recovery values from 96.0 to 104.3%.

## 1. Introduction

3-hydroxy-4-[(4-sulfo-1-naphthalenyl)azo]-2,7-naphthalenedisulfonic acid trisodium salt, amaranth, belongs to synthetic pyrazolone colorants [1]. Some characteristics such as persistent stability and low cost made it an appropriate candidate to form red color in several sweets, drinks, and syrups [2,3]. Despite the presence of the azo functional group and structure of aromaticity, the excessive consumption of amaranth can be associated with human health side effects, including anxiety, allergies, dizziness, and even cancer. Accordingly, the United States have recognized the ban on the use of amaranth in food, and China has allowed the use of this colorant with a maximum acceptable range of 0.05 g/kg in soft drinks [4,5]. In fact, it should be concluded that achieving a fast and sensitive analytical approach is a basic need in the detection of amaranth in different food products.

Amaranth has been detected by different techniques, including thin layer chromatography (TCL) [6], high-performance liquid chromatography (HPLC) [7], liquid chromatography-mass spectrometry (HPLC-MS) [8], spectrophotometery [9,10], capillary electrophoresis (CE) [11], and electrochemical methods [12,13,14]. Some specific problems of these methods including the time-consuming extraction processes and expensive instrumentation, complex analysis processes, and the presence of interferences have limited their applications. Electrochemical methods are gaining popularity due to their special properties, including simplicity, rapid response, simple equipment, ease of use, and cost-effectiveness compared with certain analytical techniques [15,16,17,18,19,20,21]. In addition, the reason for the increasing attention toward the modification of such electrodes can be attributed to the poor function of common electrodes in the detection of analytes [22,23,24,25,26,27,28,29,30,31]. The findings documented that the electron transfer rate can be increased and the overpotential can be significantly reduced through electrode surface modification.

Recently, nanomaterials have been widely used in various fields due to their exceptional properties [32,33,34,35,36,37,38,39]. The use of nanomaterials improves electrocatalytic properties and increases electrochemically active surface areas [40,41,42,43,44,45,46,47,48]. Graphene is a 2D nanostructure containing one or more sheets composed of carbon atoms. Therefore, it is one of the appropriate candidates to fabricate sensitive and selective sensors because of special features, including great electrical conductivity, high mechanical and thermal properties, and high specific surface area [49]. According to exceptional electronic potentials, graphene-based electrodes are able to enhance the electron transfer rate, suggesting a novel approach to develop electrochemical biosensors and sensors [50,51,52].

More recently, semiconductor-like properties can be achieved by chemically doped graphene, and charge conductivity and chemical reactivity can be enhanced by N-/P-doped graphene [53]. Nitrogen is one of the dopants with unique properties such as atomic radius close to that of carbon [54]. The electronic band structure can be controlled by doped N atoms. Moreover, mechanical strength, greater chemical stability, and special electronic properties have been reported for the N-doped graphene sheets [55] as a new material with various electrochemical purposes, such as the fabrication of sensors [56,57,58,59].

Thus, in this work, we developed a new electrochemical sensor to detect amaranth, in which a GCE was modified with N-rGO. The proposed method has advantages such as high linear dynamic range, high sensitivity, and a low limit of detection. Furthermore, this sensor successfully detected amaranth in real samples.

## 2. Experimental Section

### 2.1. Instruments and Chemicals

All electrochemical determinations were recorded by means of an Autolab potentiostat/galvanostat (PGSTAT-302N, Eco Chemie, Utrecht, The Netherlands). We exploited a single component three-electrode cell with a platinum auxiliary electrode and an Ag/AgCl (3 M KCl) reference electrode to perform the measurements. We used N-rGO/GCE as the working electrode while a Metrohm 827 pH meter (Metrohm AG, Herisau, Switzerland) was exploited to control the pH of the solutions. Analytical grade chemicals such as amaranth and other reagents were from Sigma-Aldrich and used as received.

### 2.2. Synthesis of N-rGO Sheets

The N-rGO sheets were prepared by a simple hydrothermal method. Firstly, exfoliated GO (100 mg) was dispersed by ultrasonication in deionized water (100 mL), and the pH of the above solution was adjusted to pH = 10 by dropwise adding ammonia solution (NH_3_·H_2_O (wt. 30%)). Next, urea (6.0 gr) was added and ultrasonicated for three hours. Then, the prepared solution was kept in an autoclave reactor at 180 °C for 12 h. After the autoclave reached ambient temperatures (25 °C), the samples were separated by centrifuge, washed several times by using deionized water, and its pH was adjusted to neutral. At last, the products (N-rGO sheets) were freeze-dried.

### 2.3. Preparing the Electrode

The synthesized N-rGO sheets were used for the modification of a *GCE* to prepare N-rGO/GCE. For this purpose, N-rGO suspension (1 mg/mL) in deionized water was prepared, and 4 μL of the N-rGO suspension was drop casted onto the GCE surface and allowed to dry completely in ambient conditions.

## 3. Result and Discussion

### 3.1. Characterization of N-rGO Sheets

**Figure 1:**
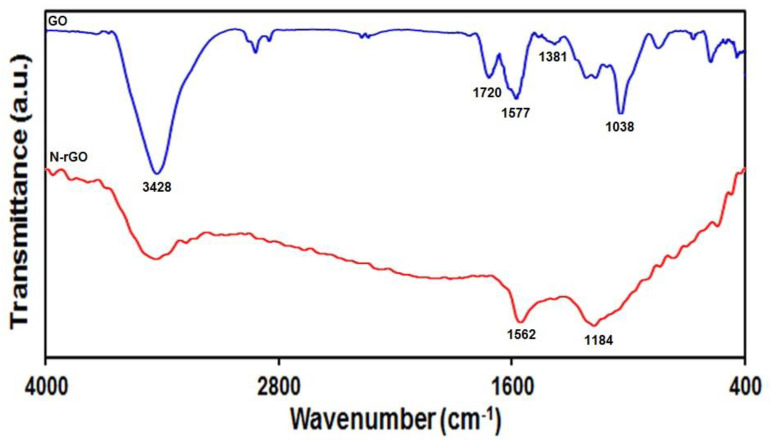
FT-IR spectrum of GO and N-rGO.

A comparison of the FT-IR spectrum of GO and N-rGO is given in Figure 1. In the characteristic spectrum of GO, various functional groups in the structure include the vibration modes of O-H (3428 cm^−1^), C=O (1720 cm^−1^), C=C (1577 cm^−1^), phenolic C-O (1381 cm^−1^), and epoxy C-O-C (1038 cm^−1^). These index peaks indicate that there are several oxygen-containing functional groups (hydroxyl, epoxy, and carboxyl) on the surface of GO. Moreover, for N-rGO, the absence of absorption peaks of oxygen groups indicated that GO has been effectively reduced during the hydrothermal treatment. In addition, there are two peaks at 1562 cm^−1^ and 1184 cm^−1^, which can be attributed to C=N (sometimes C=C and C=N bonds can stretch at the same wavelength) and C-N. This result confirmed that Gr was successfully N-doped. The FT-IR spectrum of the as-prepared sample was consistent with previous reports [60,61,62].

**Figure 2:**
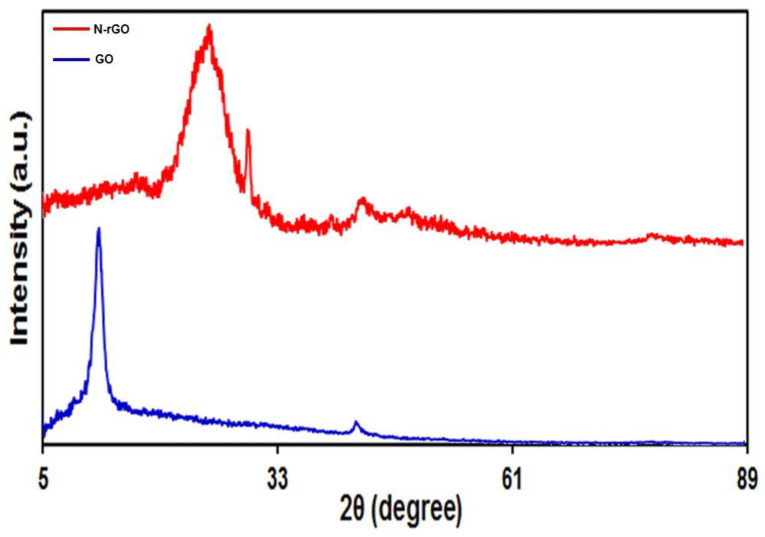
XRD pattern of GO and N-rGO.

GO and N-rGO were characterized by XRD (Figure 2). The XRD pattern of GO depicts a sharp absorption peak at 2θ = 11.5, with an inter-planer space of 7.69 Å. GO possesses a larger inter-planer space compared to graphite due to the presence of H_2_O molecules and various oxygen groups on its surface. In comparison, the XRD pattern of N-rGO confirmed the high reduction degree, as depicted in Figure 2. After hydrothermal process and in addition to the nitrogen-doping process, the peak at 2θ = 11.5 disappeared, pointing to the successful reduction of GO. However, a broad absorption peak at around 24.7° appeared in N-rGO pattern [63], which can be attributed to an inter-planar distance of 3.602 Å, proving the existence of π–π stacking between Gr layers [64].

**Figure 3:**
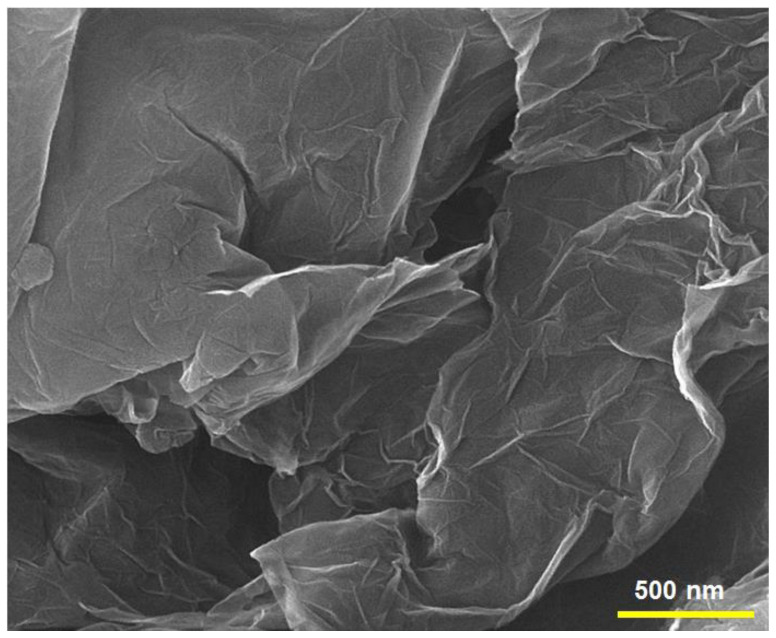
The FE-SEM image of N-rGO sheets.

The FE-SEM image of N-rGO, presented in (Figure 3) showed that N-rGO still maintain the 2D ultrathin flexible structure of the pristine Gr. Moreover, rGO sheets showed a wave structure that was similar to a thin, wrinkled paper. These results demonstrated the efficient N-doped process for the Gr. The FE-SEM image of as-prepared sample was consistent with previous reports [63,65,66].

**Figure 4:**
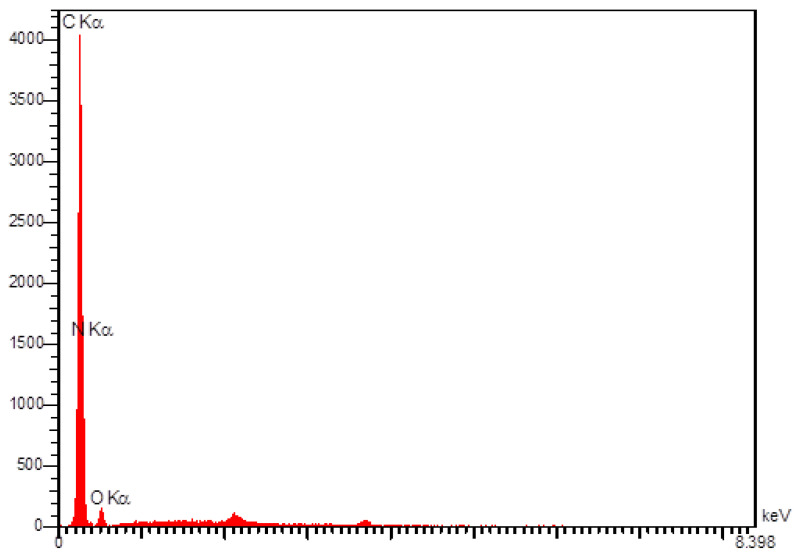
The EDS analysis of N-rGO.

We exploited energy dispersive X-ray spectroscopy (EDS) techniques for the analysis of elemental compositions (Figure 4). The EDS analysis showed that N-rGO sheets contain C, N, and O elements. This result demonstrated that the efficient N-doped process for rGO.

### 3.2. Electrochemical Behaviour of Amaranth at the Various Surface of Electrodes

The obtained results indicate that amaranth electro-oxidation occurred through electron and proton exchange. Furthermore, the optimization of the pH value in detecting the analyte was necessary. We used the DPV technique to explore the electrochemical response of amaranth on the N-rGO/GCE surface in phosphate buffer solution (PBS, 0.1 M) for pH ranging from 2 to 9. What is noteworthy is that the amaranth electro-oxidation on the N-rGO/GCE surface, in neutral conditions, was higher than in alkaline or acidic medium. Accordingly, the value of 7.0 was chosen as the optimal pH for amaranth electro-oxidation on the N-rGO/GCE’s surface.

**Figure 5:**
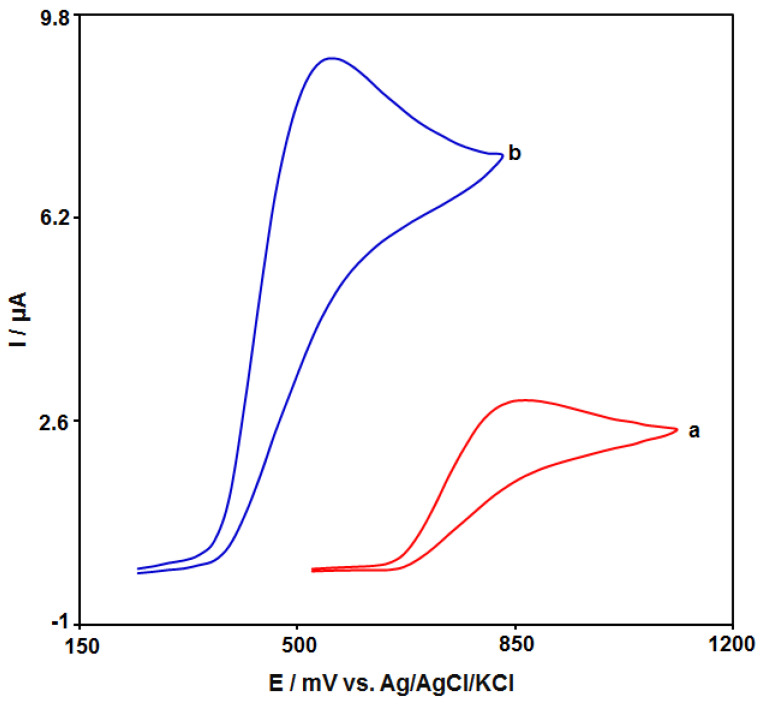
Cyclic voltammograms captured for N-rGO/GCE (**a**) and bare GCE (**b**) in PBS (0.1 M) at the optimized pH value of 7.0 in exposure to 100.0 μM of amaranth at 50 mV/s scan rate.

The electrochemical performance of N-rGO/GCE in comparison with bare GCE was studied by the CV technique at exposure to 100.0 μM of amaranth at 50 mV/s in PBS (0.1 M). The CVs of all as-fabricated electrodes in this study can be observed in Figure 5. The N-rGO/GCE voltammetric behavior (curve b) exhibited the relatively strongest and most characteristic anodic peak at 560 mV, sequentially. The bare GCE voltammetric behaviour (curve b) exhibited a relatively weak oxidation peak with less intensity at 860 mV, sequentially. Consequently, N-rGO/GCE has obviously better electrocatalytic behaviour than the bare GCE towards the amaranth with the relatively strong current response.

### 3.3. The Effects of the Scan Rate

**Figure 6:**
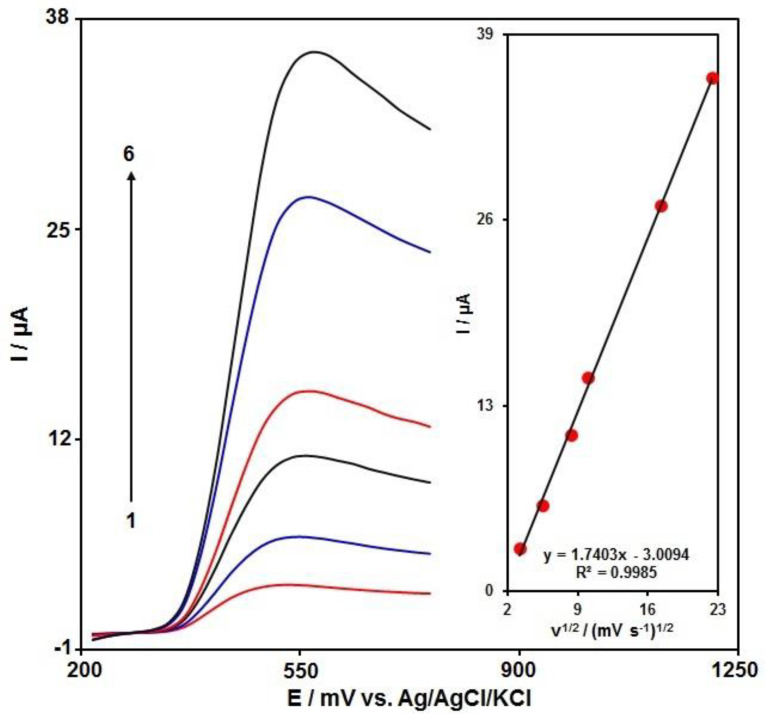
LSVs captured for N-rGO/GCE in PBS (0.1 M) at the optimized pH value of 7.0 in exposure to 100.0 μM of amaranth at various scan rates; (1): 10, (2): 30, (3): 70, (4): 100, (5): 300, and (6): 500 mV/s, sequentially; inset: changes in anodic peak currents against ν^1/2^.

We investigated the effect of the scan rate on the amaranth oxidation peak to analyze the electrode processes. We recorded the linear sweep voltammograms (LSVs) for 100.0 μM of amaranth in PBS (0.1 M) at different scan rates. We found a linear dependence of the anodic peak currents on the square root of the scan rate (Figure 6). Such a finding led us to identify the diffusion-controlled process as the process responsible for amaranth oxidation on N-rGO/GCE.

### 3.4. Chronoamperometric Measurements

**Figure 7:**
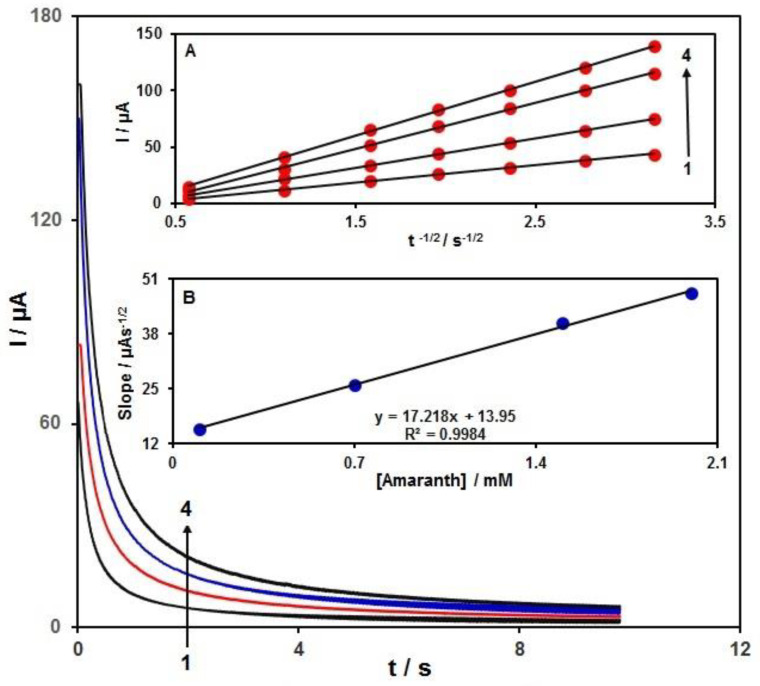
Chronoamperograms for N-rGO/GCE in PBS (0.1 M) at the optimized pH 7.0 for different amaranth contents; (1): 0.1, (2): 0.5, (3): 1.5, and (4): 2.0 mM of amaranth; insets: (A) I against t^−1/2^ plots from chronoamperograms 1 to 4. (B) Plot of straight line slope versus amaranth content.

Chronoamperometric analysis (Figure 7) is applied to calculate the diffusion coefficient of amaranth. As displayed in Figure 7 (A), the I vs. t^−1/2^ plots have been utilized for the optimal fits of different amaranth contents in PBS (0.1 M). Using various amaranth contents on N-rGO/GCE, we performed chronoamperometric measurements with the working electrode at the potential of 0.6 V. The slopes of the obtained straight lines versus amaranth contents were drawn to exploit the Cottrell equation below:

I = nFAD^1/2^C_b_π^−1/2^t^−1/2^

where D (cm^2^/s) is the diffusion coefficient and C_b_ (mol/cm^3^) indicates the bulk content. There was a linearity for the I plots against t^−1/2^ under diffusion control over various amaranth contents. We used the slope vs. amaranth content plot (Figure 7 (B)) to extract the mean D value for amaranth, obtaining 2.5 × 10^−5^ cm^2^/s.

### 3.5. Calibration Plot and LOD

**Figure 8:**
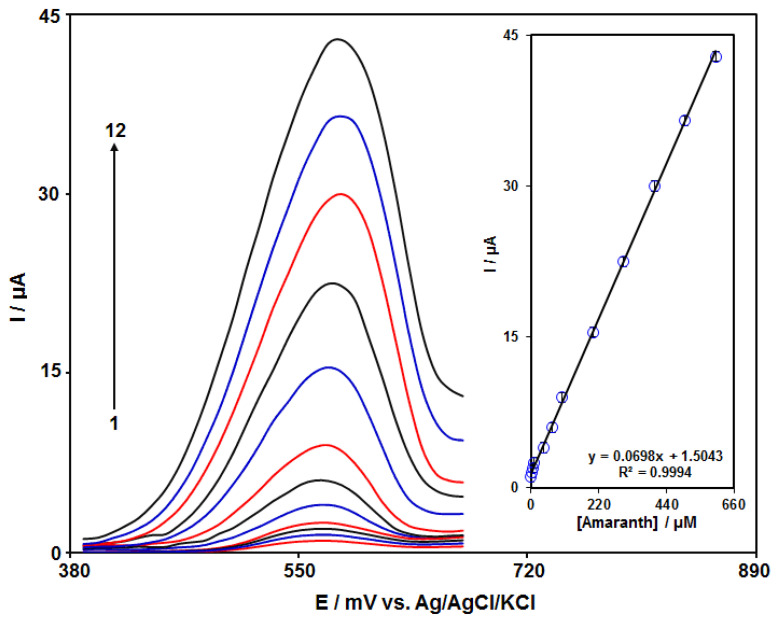
DPVs captured for N-rGO/GCE in PBS (0.1 M) at the optimized pH 7.0 in exposure to various amaranth contents; (1): 0.1, (2): 1.0, (3): 5.0, (4): 10.0, (5): 40.0, (6): 70.0, (7): 100.0, (8): 200.0, (9): 300.0, (10): 400.0, (11): 500.0, and (12): 600.0 µM of amaranth; inset: peak current versus various amaranth contents (0.1–600.0 µM).

Amaranth content was measured by the DPV technique. The DPVs captured for N-rGO/GCE at various amaranth contents in PBS (0.1 M) are shown in Figure 8. There was a stepwise enhancement in the amaranth oxidation current by gradually increasing amaranth contents, meaning the applicability of N-rGO/GCE for electrochemically sensing the amaranth. Figure 8 (inset) represents the alterations in the oxidation signal on N-rGO/GCE as a function of various amaranth contents (0.1–600.0 µM), having a low LOD of 0.03 μM. In addition, Table 1 shows that the N-rGO/GCE can compete with other sensors for the determination of amaranth.

### 3.6. Analysing the Real Sample

The ability of N-rGO/GCE for sensor applications in the detection of amaranth was determined in real specimens of tap water, apple juice, and orange juice according to the standard addition method, as seen in Table 2. The recorded recovery rates ranged from 96.0% to 104.3%, thereby highlighting the appreciable applicability of N-rGO/GCE in sensing amaranth in real specimens.

## 4. Conclusions

In this study, we developed a novel sensitive electrochemical sensor, based on N-rGO sheets, for the detection of amaranth. The proposed sensor exhibits remarkable electrocatalytic activity and excellent sensitivity toward amaranth detection with a linear response over a wide concentration range (0.1–600.0 µM) and a low detection limit (0.03 µM). Moreover, the applicability of N-rGO modified GCE was tested in real samples with good accuracy and satisfying recovery.

## Figures and Tables

**Table 1:** Linear range and LOD obtained at the N-rGO/GCE for the determination of amaranth compared with other sensors.

Electrochemical Sensor	Method	Linear Range	LOD	Ref.
Fe_3_O_4_@reduced graphene oxide/GCE	DPV	0.05–50 ìM	50 nM	[2]
Ordered mesoporous carbon/GCE	DPV	1.0 × 10^−7^–3.0 × 10^−6^ M	3.2 × 10^−8^ M	[3]
Single-walled carbon nanotube-titanium nitride nanocomposite/GCE	DPV	0.1–100 ìM	40 nM	[67]
Multi-wall carbon nanotube thin film/GCE	DPV	40 nM–0.8 ìM	35 nM	[68]
Co_3_O_4_-CeO_2_/graphene nanocomposite modified electrode	DPV	2–96 μM	0.1591 μM	[69]
N-rGO/GCE	DPV	0.1–600.0 µM	0.03 μM	This Work

**Table 2:** The application of N-rGO/GCE for the determination of amaranth in tap water, apple juice, and orange juice. (*n* = 5).

Sample	Spiked (µM)	Found (µM)	Recovery (%)	R.S.D. (%)
Tap Water	0	-	-	-
	5.0	4.9	98.0	3.2
	6.0	6.2	103.3	1.7
	7.0	7.1	101.4	2.4
	8.0	7.8	97.5	2.6
Apple Juice	0	-	-	-
	4	4.1	102.5	1.7
	6	5.8	96.7	3.5
	8	8.1	101.2	2.6
	10	9.9	99.0	2.7
Orange Juice	0	-	-	-
	5	4.8	96.0	3.6
	7.0	7.3	104.3	2.8
	9.0	9.1	101.1	3.0
	11.0	10.9	99.1	1.9

## Data Availability

The data presented in this study are available upon request from the corresponding authors.
